# Chronic Myeloproliferative Neoplasms: a Collaborative Approach

**DOI:** 10.4084/MJHID.2010.017

**Published:** 2010-06-21

**Authors:** Lisa Pieri, Paola Guglielmelli, Alessandro M. Vannucchi

**Affiliations:** Unità Funzionale di Ematologia, Dipartimento di Area Critica, Università degli Studi, Istituto Toscano Tumori, Firenze

## Abstract

The classic chronic myeloproliferative neoplasms (MPN) include different entities that pose significant challenges for their optimal diagnosis, treatment and overall management. Polycythemia Vera and Essential Thrombocythemia are the most common among chronic myeloproliferative neoplasms (MPNs); major causes of morbidity and mortality are represented by arterial and venous thrombosis, as well as evolution to myelofibrosis or transformation to acute leukemia. However, survival is only minimally affected. Therapy aims at reducing the rate of thrombosis without increasing the risk of hematologic transformation which could be caused by exposure to cytotoxic drugs. On the other hand, survival is significantly reduced in primary myelofibrosis, and the clinical manifestations may be disabling. In the absence of therapies with the potential of curing the disease, a careful risk-oriented approach is employed for stratifying patients to the most appropriate, currently available, therapeutic options. In this brief review, we will discuss some of the key issues that can arise along the clinical course of MPNs and require an integrated, strictly patient-oriented, approach.

## Introduction:

The approach to a patient suffering from a Philadelphia-chromosome negative, classic Myeloproliferative Neoplasm (MPN)^1^ can be mostly of a strict hematological pertinence or can require a complex, multidisciplinary approach in cases where the disease has an aggressive course or presents or evolves with complications (Table 1). There are obvious differences in clinical presentation that are related to the unique MPN entities, the worse being primary myelofibrosis (PMF); however, it must be kept in mind that protean clinical issues are largely and often unpredictably patient-related. However, from a clinical point of view, the most challenging consideration for the physician is that while the overall survival of PV or ET patients may be only marginally affected by the disease, survival may range from a few months to an excess of a decade in PMF patients. The main complications of polycythemia vera (PV) and essential thrombocythemia (ET) are cardiovascular in nature and constitute the leading cause of death.^2^ As such, they may require counseling of specialists from different fields including cardiologists, vascular surgeons, gastroenterologists (for the manifestations and complications of splancnic vein thromboses), radiologists, and experts in anticoagulation therapy.^3^ A young lady with ET who plans to become pregnant, or is expected to deliver, poses specific problems in antithrombotic treatment during pregnancy and in the immediate post-delivery period, as well as requires careful obstetrician management.^4^ Furthermore, opportunity about a myelosuppressive treatment before conception and during pregnancy should be accurately discussed in case a woman is considered at high-risk because of the characteristics of her disease or a history of previous abortions. Therapeutic decisions regarding massive and symptomatic splenomegaly in MF, and more infrequently in PV or ET, may involve the hematologist, the surgeon, as well the radiotherapist.^5^ Leukemic transformation is associated with a very dismal outcome with conventional chemotherapeutic approaches because of intrinsic characteristics of the disease as well as the almost universal presence of comorbidities in older patients; therefore, decisions about choice between intensive chemotherapy, a smoother therapeutic approach, supportive treatment only, as well as counseling for the end-of-life decisions or hospice treatment need to be taken by discussing with the patients, relatives and colleagues from different medical specialites.^6^ Cutaneous complications from cytotoxic therapy with hydroxyurea (leg ulcers) represent a relatively common side effect of therapy and constitute a therapeutic dilemma that requires dermatologist’s approach, nurse’s care, and eventually additional specialists (experts in hyperbaric therapy, reconstructive surgeon). Recent improvement in the use of hematopoietic stem cell transplantation in patients with MF, and the availability of novel drugs, that include JAK2 inhibitors,^7^ inhibitors of histone deacethylases^8^ and possibly others, will probably result in a modification of our current approach to MPNs in the near future, making the management of these disorders even more complex but, hopefully, more effective possibly leading to a cure.

In this review we will briefly discuss four issues that are: genetic counseling, the management of pregnancy in a woman affected by MPN, how to face a massively enlarged spleen in myelofibrosis, and the criteria for choosing between conventional and experimental therapy.

## Genetic counseling:

MPNs are not hereditary disorders, and as such there has been no indication until now to refer patients to an expert of human genetics. However, the fact that familial forms of MPNs, whose existence has been long known, are discovered more and more frequently (their rate probably being linearly dependent on the accuracy the existence of MPNs in the family is searched for by the referring hematologist), and recent discoveries about JAK2 46/1-GGCC predisposing haplotype, combined with the diffusion of these information through specialized and non-specialized web sites or the print, have all increased awareness by the patients, especially youngest. So, we can expect that the question *“..will I transmit the disease to my sons?*” is increasingly proffered to physician by newly diagnosed MPN patients. At this time speaking, MPNs continue to be purely acquired disorders, with no evidence of germline transmission, no need of genetic counseling, and no need, on the hematologist’s side, to suggest testing familial members with either simple (automated blood cell count) or complex (molecular analysis of JAK2 or similar mutations) laboratory approaches. It is key to avoid generating unjustified and unethical “mind storms” in the patients and their relatives by an inappropriate usage of the novel information deriving from our increasing knowledge of the molecular complexity of MPNs.

## How to manage pregnancy:

The opportunity for a physician to deal with a pregnant woman is definitely more common in ET than in PV due to younger age. There is no controlled study concerning management strategies, and recommendations only reflect experts’ opinions.^9^,^10^ About seventy per cent of 461 pregnancies reported in ET women ended with successful live.^11^ On the other hand, the incidence of major maternal complications was significantly higher (44%) in the 18 PV patients included in one single-centre study^12^ as compared to ET (about 5%). Interestingly, an association of *JAK2*V617F mutational status with greater occurrence of pregnancy complications in ET has been described.^13^ It is key to identify a “at high-risk pregnancy”, such as in a woman who had history of previous thrombosis or presented severe complications during previous pregnancies. The latter include 3 or more first-trimester or 1 or more third-trimester losses; a previous birth weight less than the 5^th^ centile of gestation; development of symptoms of pre-eclampsia; intrauterine death or stillbirth. In fact, a “low-risk pregnancy” in ET could be managed with observation only a part for maintaining hematocrit level lower than 45% in a few cases where the physiologic decrease of hemoglobin levels does not happen. There is no clear indication to support prophylactic low-dose aspirin in the absence of microcirculatory symptoms in ET, while all patients with PV should receive aspirin during pregnancy until about 1 week before planned delivery. However, in absence of controlled studies, some authors consider appropriate the administration of low-dose aspirin even in “low risk” ET. The presence of classic cardiovascular risk factors in these patients, such as obesity or bed rest, can be a issue for choosing LMWH prophylaxis instead of aspirin. The lack of obvious correlation between thrombocytosis and pregnancy complication makes correction of platelet count not a relevant target for therapy, also considering that platelet count often reaches and maintains normal levels throughout pregnancy. When there is the need to control myeloproliferation interferon-alpha is the only drug recommended because its lack of teratogenic properties. Neither HU nor anagrelide can be used during pregnancy. In the absence of any evidence-based information in the literature, and only as a cautionary attitude, we suggest at least a six-month period wash-out from either drug in case of planned conception; however, it should be stressed that fetal abnormalities have been reported only in one out of 60 pregnancies that were successfully completed under HU.^12^ Low molecular weight heparin (LMWH) is recommended after delivery until six weeks postpartum to prevent venous throemboembolism; eventually, prophylactic LMWH could be started 1–2 weeks before expected labour and stopped about 12 hours before. Some experts also consider the combined use of aspirin and LMWH throughout pregnancy and for six weeks after delivery in high-risk ET patients and in all PV women.^10^ Thus, the management of pregnancy is a MPN woman is a complex issue that requires interactions of the hematologist with the couple to counsel about the best approach and to reassure the woman about potential risks and benefits of no therapy versus therapy; and with the gynecologist and obstetrician for an accurate follow-up (it should not be undermined that this is anyway a “at risk” pregnancy compared to control population). We have to reassure the woman who wants to become a mother that this is feasible and low-risk, and do our best to make her feeling helped and safe at any time point during pregnancy and delivery. There are also some grey areas strictly connected with the issue of pregnancy in MPNs; these concern counseling about hormonal therapy for contraception (that should be discourages as possible), about the risk of fetal defects in the case conception happened whilst on therapy, and finally also about the potential risks associated with the use of cytotoxic drugs by the father. Unfortunately, there is little to no experience at this regard that can help making recommendations, so that only prudent, individualized and shared approach can be employed.

How to face massive splenomegaly in myelofibrosis: Splenomegaly can be found in all the MPNs, but it is a main complaint of patients with myelofibrosis as the manifestation of massive extramedullary hematopoiesis that results largely from the substitution of hematopoietic tissue with reticulin and collagen fibers in the bone marrow. Splenomegaly causes local problems (early saxiety, manifestations due to local compression on organ and vessels) and contributes to systemic symptoms, particularly cachexia, and hematological abnormalities due to sequestration of red blood cells, granulocytes and platelets. An excellent critical review of how to manage splenectomy has been published recently.^5^ No conventional drug is effective against huge spleen enlargement, although hydroxyurea may help in the early phase of disease. Splenectomy carries a considerable risk during surgery and in the post-surgery phase due to vascular complications, hemorrhages, infections, and myeloproliferative reactions (**Table 2**). Splenomegaly can also represent a negative prognostic factor for patients undergoing hematopoietic stem cell transplantation.^14^ Thus, managing massive splenomegaly in myelofibrosis requires an integrated experienced team involving the referring hematologist, the radiotherapist, the surgeon, a physician experienced in managing splancnic thrombotic complications after surgery, and eventually an hematologist with experience in transplanting myelofibrosis patients. But, first of all, a comprehensive discussion with the patient of the indications, risk and expected benefits and possible risks is mandatory.

## Choosing between conventional and experimental therapies:

The management of ET and PV has been traditionally problematic because of the lack of robust evidence-based management guidelines. Factors for identifying high-risk patients who deserve a more aggressive treatment have been discovered and validated in large clinical studies, and novel ones are under strict scrutiny more recently.^1^ The former are represented by older age and a previous history of vascular events, the latter are the JAK2V617F mutational status or burden and leukocytosis. The widely employed drug in high risk patients is hydroxyurea in association with anti-platelet agents; the approach successfully reduces (but does not eliminate) the risk of major thrombosis, but whether it is able to modify the course of disease is questionable. Although the rate of leukemia transformation is probably not different from control patient populations, use of a cytotoxic drug is preferably to avoid in younger patients. Also, although the rate of resistance/intolerance to hydroxyurea in probably less than10% of all patients, in some instances the treatment has to be shifted towards other cytotoxic agents, with a measurable increase in the risk of leukemic transformation. The scenario in therapeutic armamentarium for MPNs is dramatically changing (**Table 3**), and in the next few years it will be possible that a number of clinical trials will be activated hopefully providing new avenues for improving the outcome, the quality of life, and reducing complications associated with these disorders.

## Figures and Tables

**Table 1.** Some of the key issues in the management of MPN patients that require integrated skills.

**Polycythemia Vera / Essential Thrombocythemia**	**Myelofibrosis**
Prevention of vascular complication Ongoing treatment of prior thrombotic events Control of myeloproliferation Control of splenomegaly Symptomatic control of disease manifestations	Treatment of anemia and cytopenias Control of splenomegaly Control of myeloproliferation Prevention of vascular complication Symptomatic control of disease manifestations Palliative therapy versus HSCT
Familial forms and discussion of genetic predisposition Pregnancy management and counseling Choice between no therapy, conventional therapy, experimental therapy	

**Table 2.** Indications and outcome of splenectomy for massive splenomegaly in myelofibrosis. Numerical values indicate proportion of the patients. From Mesa^5^.

**Indications** Symptoms ascribable to local compression (50%) Refractory, transfusion-dependent anemia (25%) Refractory thrombocytopenia (15%) Portal hyperthension (10%)
**Complications of surgery** Overall morbidity (30–40%) Hemorrhage (15%) Thrombosis (10–15%) Fatalities (7–10%)
**Improvement rate** Symptoms ascribable to local compression: 50% Anemia: 50% Thrombocytopenia: 30% Portal hyperthension: 40%
**Long-term outcome** Leukemia: 10–15% Progressive hepatomegaly: 10–15%

**Table 3.** Novel drugs on the pipeline for MPNs.

**Drug class**	**Molecules**	**Main targets / disease**	**References**
JAK2 inhibitors	INCB 018424 TG101348	Splenomegaly, constitutional symptoms (mainly MF, potentially PV or ET)	[15] [16]
Immunomodulating agents	Lenalidomide, Pomalidomide	Anemia (MF)	[17] [18]
Interferons	PEG-interferon-a	Mainly PV and ET, preliminary data in early MF	[19]
Histone deacethylase inhibitors	Givinostat	Mainly PV	[20]
